# Mitochondrial iron and energetic dysfunction distinguish fibroblasts and induced neurons from pantothenate kinase-associated neurodegeneration patients

**DOI:** 10.1016/j.nbd.2015.02.030

**Published:** 2015-09

**Authors:** Paolo Santambrogio, Sabrina Dusi, Michela Guaraldo, Luisa Ida Rotundo, Vania Broccoli, Barbara Garavaglia, Valeria Tiranti, Sonia Levi

**Affiliations:** a San Raffaele Scientific Institute, Division of Neuroscience, 20132 Milano, Italy; b Molecular Neurogenetics Unit, Foundation IRCCS-Neurological Institute “Carlo Besta”, 20126 Milano, Italy; c University Vita-Salute San Raffaele, 20132 Milano, Italy

**Keywords:** DCF, dichlorofluorescein, DFO, deferoxamine, DHR-123, dihydrorhodamine 123, FAC, ferric ammonium citrate, iNs, induced neurons, IRP1, iron regulatory protein 1, LIP, labile iron pool, NBIA, neurodegeneration with brain iron accumulation, PKAN, pantothenate kinase associated neurodegeneration, PIH, pyridoxal isonicotinoyl hydrazone, ROS, reactive oxygen species, RPA, rhodamine B-[(1,10-phenanthroline-5-yl)-aminocarbonyl]benzyl ester, RPAC, rhodamine B-[(phenanthren-9-yl)-aminocarbonyl]-benzylester, TMRM, tetramethylrhodamine methyl ester, Iron metabolism, Mitochondria, Reactive oxygen species, NBIA, PANK2, Induced neurons

## Abstract

Pantothenate kinase-associated neurodegeneration is an early onset autosomal recessive movement disorder caused by mutation of the pantothenate kinase-2 gene, which encodes a mitochondrial enzyme involved in coenzyme A synthesis. The disorder is characterised by high iron levels in the brain, although the pathological mechanism leading to this accumulation is unknown. To address this question, we tested primary skin fibroblasts from three patients and three healthy subjects, as well as neurons induced by direct fibroblast reprogramming, for oxidative status, mitochondrial functionality and iron parameters. The patients' fibroblasts showed altered oxidative status, reduced antioxidant defence, and impaired cytosolic and mitochondrial aconitase activities compared to control cells. Mitochondrial iron homeostasis and functionality analysis of patient fibroblasts indicated increased labile iron pool content and reactive oxygen species development, altered mitochondrial shape, decreased membrane potential and reduced ATP levels. Furthermore, analysis of induced neurons, performed at a single cell level, confirmed some of the results obtained in fibroblasts, indicating an altered oxidative status and signs of mitochondrial dysfunction, possibly due to iron mishandling. Thus, for the first time, altered biological processes have been identified in vitro in live diseased neurons. Moreover, the obtained induced neurons can be considered a suitable human neuronal model for the identification of candidate therapeutic compounds for this disease.

## Introduction

Neurodegeneration with brain iron accumulation (NBIA) is a heterogeneous group of genetic disorders characterised by radiological evidence of focal accumulation of iron in the brain, usually in the basal ganglia and extrapyramidal dysfunction (Schneider et al., 2013, Levi and Finazzi, 2014). These disorders are characterised by early or late onset, with the main symptoms associated with problems in movement, spasticity and cognitive impairment. Approximately 50% of cases of NBIA can be explained by mutations in the *PANK2* gene that cause an autosomal–recessive form of the disease, termed pantothenate kinase-associated neurodegeneration (PKAN or NBIA type I; OMIM 234200) (Hayflick, 2014). Magnetic resonance imaging (MRI) is particularly useful for distinguishing the cases of PKAN from other NBIA forms. In the majority of PKAN patients, the T2-weighted images show a hyperintense lesion of the globus pallidus, surrounded by a hypointense area (Angelini et al., 1992). This combination of hyper- and hypointense areas in the globus pallidus gives rise to a pattern defined as the “eye of the tiger”, which is almost pathognomonic of the disease. The brain regions in which iron accumulates at pathological levels are the globus pallidus and substantia nigra, where iron-positive spheroidal bodies are visible, usually in the vicinity of swollen axons. Other neuropathological signs include demyelination, neuronal loss and gliosis (Kruer et al., 2011).

*PANK2* codes for the pantothenate kinase-2 (PANK2), a mitochondrial enzyme that catalyses the first limiting step of the de novo biosynthesis of coenzyme A (CoA). CoA is a key factor in several cellular processes, including mitochondrial energy metabolism, anabolism and catabolism of fatty acids, as well as protein biosynthesis (Leonardi et al., 2005). In humans, 4 genes code for pantothenate kinases, but only PANK2 has mitochondrial localisation. Mutations in *PANK2*, which is located on chromosome 20, are spread over all 7 exons and include missense, nonsense, frameshift and splicing site mutations (Hartig et al., 2006). Mutations in *PANK2* result in enzyme deficiency, leading to insufficiency of the final product and accumulation of upstream substrates, such as N-pantotenoil-cysteine and pantetheine, which are potentially toxic (Leoni et al., 2012). In particular, cysteine is a potent iron chelator, and it has been proposed that high local levels of cysteine are the basis of the subsequent accumulation of iron, resulting in increased oxidative stress (Perry et al., 1985). The other hypothesis to explain the observed iron accumulation suggests that alterations in phospholipid metabolism due to CoA-deficiency may injure the membranes, with consequent oxidative stress that leads to iron dys-homeostasis (Leonardi et al., 2007). Different animal disease-models have been developed using *Drosophila melanogaster* and *Mus musculus*. *Drosophila* PKAN models partially recapitulate the human phenotype, showing locomotor dysfunction and neurodegeneration (Rana et al., 2010). In addition, a *Pank2* KO mouse model does not fully recapitulate the human phenotype (Kuo et al., 2005), unless particular dietary conditions are used (Brunetti et al., 2014). The double knock-out of more than one *Pank* in mice produces a very drastic phenotype resembling a metabolic syndrome (Garcia et al., 2012). However, these models do not show iron accumulation in the brain and are not useful for studying the pathogenetic mechanism leading to iron imbalance, which is hallmark sign in the brains of patients. Two in vitro studies on cellular models have attempted to explain the relationship between *PANK2* deficiency and iron deregulation. In HeLa, HepG2 and SH-SY5Y cells, the specific siRNA silencing of *PANK2* affects cell proliferation, induces cellular iron deficiency and increases the expression of the iron exporter ferroportin (Poli et al., 2010). PKAN fibroblasts, maintained in chronic iron supplementation, showed disturbed iron sensor protein iron regulatory protein 1 (IRP1) activity, resulting in deregulation of ferritin and transferrin receptor1 (TfR1), as well as a larger intracellular bioactive labile iron pool (LIP). This effect results in higher reactive oxygen species (ROS) development and leads to increased cellular oxidative status (Campanella et al., 2012).

Mitochondria are the main sites of iron utilisation in the cell (Levi and Rovida, 2009). This organelle employs the metal to sustain the biosynthesis of the iron sulphur cluster (ISC) and heme cofactors, which are prosthetic groups of widespread proteins involved in key biological processes, such as electron transfer, DNA synthesis and repair, metabolic and regulatory processes (Stehling et al., 2014). Thus, mitochondria play a central role in cell life, not only for energy supply but also for cellular iron handling. This important role is highlighted by the fact that defects in mitochondrial iron homeostasis lead to pathological phenotypes and cell death (Levi and Rovida, 2009). Here, we evaluated mitochondrial functionality in terms of iron handling and energetic profile to investigate whether the iron-dependent oxidative status alteration, previously revealed in PKAN patients' fibroblasts (Campanella et al., 2012), also affects the mitochondrial compartment. Furthermore, by taking advantage of the recently developed technology (Amamoto and Arlotta, 2014, Caiazzo et al., 2011) that allows neurons to be directly transdifferentiated from fibroblasts, we generated induced neurons (iNs) from PKAN patients to establish a suitable disease model in which to study the consequences of *PANK2* dysfunction.

## Material and methods

### Cell culture

We used primary skin fibroblasts from three unaffected subjects (controls 1, 2 and 3, two neonatals and one adult) purchased from ATCC and from three PKAN patients selected from the Movement Disorders Bio-Bank available at the Neurogenetics Unit of the Neurological Institute ‘Carlo Besta’ (INCB), Milan, Italy. Two PKAN patients (marked F419fsX472(a) and F419fsX472(b), biopsy was made at the age of two and four years old) (Campanella et al., 2012) were brothers who are homozygous for the same frame shift mutation that results in a truncated PANK2 protein (F419fsX472). The third, Y190X (Hartig et al., 2006), was homozygous for a mutation that produced a truncated amino acid chain. The fibroblasts were grown in DMEM (Lonza) supplemented with 10% FBS (Lonza), 100 mg/ml streptomycin, 100 U/ml penicillin and 4 mM l-glutamine (Sigma).

### Generation of iNs from human fibroblasts by direct reprogramming

Human fibroblasts from patients and controls were grown in medium for fibroblasts (DMEM, FBS, nonessential amino acids, sodium pyruvate, and penicillin/streptomycin) plated onto Matrigel-coated 24-well plates (5 × 10^4^ cells/well). For the immune-histochemical analysis, some of these cells were plated onto Matrigel-coated glass coverslips. On the second day, the fibroblasts were infected by lentivirus in which cDNAs for transcription factors (Mash1, Nurr1, and Lmx1a) had been cloned (Caiazzo et al., 2011) under the control of a tetracycline-responsive promoter. Sixteen to twenty hours after infection, the cells were switched into fresh fibroblast medium containing doxycycline (2 mg/ml), and after a further 48 h, the medium was replaced with neuronal inducing medium (DMEM F12, 25 μg/ml insulin, 50 μg/ml transferrin, 30 nM sodium selenite, 20 nM progesterone, and 100 nM putrescine and penicillin/streptomycin) containing doxycycline (all from Sigma). The medium was changed every 2–3 days for a further 20 days.

### Immunoblotting

Soluble cellular extracts for immunoblotting were obtained by lysing cells in 20 mM Tris–HCl, pH 7.4, 1% Triton X-100, and protease inhibitor cocktail (Roche) followed by centrifugation at 16,000 *g* for 10 min. Fifteen micrograms of total proteins was separated by sodium dodecyl sulphate-polyacrylamide gel electrophoresis (SDS-PAGE), and immunoblotting was performed using specific antibodies: anti-citosolic aconitase (cAco) (Campanella et al., 2012) was used at a dilution of 1:500; anti-mitochondrial aconitase (mAco) (Antibody Verify) was used at a final concentration of 1 μg/ml; the anti-β-actin antibody (Sigma) was used at a dilution of 1:6000; the mouse monoclonal anti-PANK2 (Origene) antibody was used at a dilution of 1:2000; and the mouse monoclonal anti-SDH (70 kDa subunit) (MitoScience) was used at a final concentration of 0.1 μg/ml, followed by peroxidase-labelled secondary antibodies (Sigma-Aldrich). Band intensity was revealed by the ECL-chemiluminescence kit (GE Healthcare). The total protein contents were measured using the BCA protein assay (Thermo Fisher Scientific) calibrated with bovine serum albumin.

### Determination of aconitase activity

Aconitase activity was in-gel assayed as described in Tong and Rouault (2006). The patient and control fibroblasts were grown in DMEM, harvested, washed in PBS and lysed in 20 mM Tris–HCl buffer, pH 7.4, 1% Triton X-100, protease inhibitor cocktail, 2 mM citrate, 0.6 mM MnCl2, and 40 mM KCl. Soluble extracts (40 μg) in 25 mM Tris–HCl, pH 8.0, 10% glycerol, bromophenol blue, were loaded on PAGE gels containing 8% acrylamide, 132 mM Tris base, 132 mM borate, and 3.6 mM citrate in the separating gel; and 4% acrylamide, 67 mM Tris base, 67 mM borate, 3.6 mM citrate in the stacking gel. The run was performed at 180 V for 2.5 h at 4 °C. Aconitase activity was determined in the dark at 37 °C by incubating the gel in 100 mM Tris–HCl, pH 8.0, 1 mM NADP, 2.5 mM cis-aconitic acid, 5 mM MgCl2, 1.2 mM MTT, 0.3 mM phenazine methosulfate, and 5 U/ml isocitrate dehydrogenase. The quantification of the signal was performed using the NIH image software ImageJ.

### Determination of heme content

Heme content was measured in fibroblasts from patients and controls as previously described (Santambrogio et al., 2011). Briefly, the cells were washed with phosphate-buffered saline and dissolved in 0.25 ml of 98% formic acid and incubated for 15 min. The heme content was evaluated by analysing the clear supernatant at 400 nm, with an extinction coefficient of 1.56 × 10^5^ × M^− 1^ × cm^− 1^. The data were normalised to protein content as determined by the BioRad Protein Assay (BioRad).

### Oxidised protein detection

Oxidised proteins were detected using the OxyBlot Protein Oxidation Detection Kit (Millipore) following the manufacturer's instructions. Briefly, 10 μg of the soluble cellular extracts was derivatised to 2,4 dinitrophenylhydrazone, and 5 μg was loaded on a 12% SDS-PAGE gel, blotted and incubated with an anti-DPN antibody. The bound activity was revealed by a ECL-chemiluminescence kit (GE Healthcare).

### Glutathione measurement

Fibroblasts, treated or not with 1 mM TCEP (tris[2-carboxyethyl]phosphine) for 30 min at 37 °C, were incubated with 20 μM ThiolTracker Violet (Invitrogen) for 30 min at 37 °C, washed with PBS and fixed in 4% paraformaldehyde in PBS for 20 min at room temperature. The images were acquired by Zeiss Axiovert 135 TV fluorescence microscope, and the ThiolTracker Violet signal was quantified by ImageJ software. iNs were incubated with 20 μM ThiolTracker Violet (Invitrogen) for 30 min at 37 °C, washed with PBS and fixed in 4% paraformaldehyde in PBS for 20 min at room temperature. The cells were then permeabilised for 3 min in PBS containing 0.1% Triton X100 and 10% normal goat serum. Next, the cells were incubated with Alexa Fluor 647 mouse anti-human CD56 (N-CAM, BD Biosciences, diluted 1:40) for 1 h at 37 °C, and with 2 μg/ml Hoechst for 2 min. After washing, the cells were analysed by IN Cell Analyzer 1000 system (GE Healthcare). The ThiolTracker Violet fluorescence in N-CAM-positive cells was collected to compare relative glutathione contents.

### Determination of LIP

LIP was measured using the iron-sensitive fluorescent probe RPA (rhodamine B-[(1,10-phenanthrolin-5-yl)-aminocarbonyl]benzyl ester) (Squarix Biotechnology). Control of fluorescence probe incorporation was performed with RPAC (rhodamine B-[(phenanthren-9-yl)-aminocarbonyl]-benzylester), an analogue of RPA but without iron binding capacity and subsequent quenching. Briefly, fibroblasts were plated in 96-well plates and incubated with or without 100 μM FAC and 200 μM ascorbic acid (Sigma) for 18 h. The cells were incubated in HBSS supplemented with 10 mM glucose and 2 μM RPA or RPAC for 15 min at 37 °C. After two washes with HBSS, the cells were maintained in HBSS supplemented with 10 mM glucose. Basal fluorescence was measured using a Victor3 Multilabel Counter (Wallac, Perkin Elmer) at 530 nm (excitation) and 590 nm (emission). The quenching of RPA by iron was revealed after the addition of the specific iron chelator PIH (final concentration: 2 mM) for 30 min. The results were normalised for protein content.

### Determination of ROS

Fibroblasts were incubated with 30 μM dihydrorhodamine-123 (DHR-123, Molecular Probes) for 15 min at 37 °C and then washed with PBS and maintained in HBSS supplemented with 10 mM glucose. The fluorescence was determined using the Victor3 Multilabel Counter (PerkinElmer) at 485 and 535 nm for excitation and emission, respectively. The cells were incubated in HBSS for 30 min at 37 °C with 0.3 M H_2_O_2_, and fluorescence was determined as above.

iNs generated from fibroblasts were incubated with Alexa Fluor 647 mouse anti-human CD 56 (anti-N-CAM; BD Biosciences) for 1 h, with 20 μM of 2′,7′-dichlorodihydrofluorescein diacetate (H_2_DCFDA; Molecular Probes) for 15 min and with 2 μg/ml of Hoechst 33342 for 2 min. All of these incubations were performed at 37 °C. The cells were washed and analysed using an IN-Cell Analyzer 1000 system (GE Healthcare). The fluorescence of DCF from N-CAM-positive cells was collected to compare the relative ROS contents.

### Determination of mitochondrial membrane potential

Fibroblasts were incubated with 4 μg/ml of oligomycin (Sigma) for 1 h at 37 °C and with 2 μg/ml of Hoechst 33342 for 2 min. Then, 20 nM of tetramethylrhodamine methyl ester (TMRM; Molecular Probes) was added, and its incorporation was followed by analysis with the IN-Cell Analyzer 1000 system for 2 h. Next, 4 μg/ml of FCCP (Sigma) was added to depolarise the mitochondrial membrane, and TMRM signal followed for other 10 min.

iNs generated from fibroblasts were incubated with Alexa Fluor 488 mouse anti-human CD 56 (anti-N-CAM; BD Biosciences) for 1 h, with 20 μM of TMRM (Molecular Probes) for 15 min, and with 2 μg/ml of Hoechst 33342 for 2 min. All of these incubations were performed at 37 °C. The cells were washed and analysed by the IN-Cell Analyzer 1000 system (GE Healthcare). The fluorescence of TMRM from N-CAM-positive cells was collected to compare the relative mitochondrial membrane potential.

### Analysis of mitochondrial network and Shape Factor

The cells were plated on 35-mm glass-bottom dishes in complete DMEM. After 24 h, Mitotracker Red (Invitrogen) was added at a final concentration of 10 nM for 30 min at 37 °C. The cells were then washed three times with PBS, and a medium with 25 mM HEPES and without Phenol Red was added. Fluorescence was visualised on an Axiovert 200 epifluorescence inverted microscope (Zeiss, Germany) equipped with a 40 × fluorite objective using a CARV II Confocal Imager (BD Biosciences). The images were acquired using a CCD camera (Roper Scientific, USA). All of the imaging data were collected and analysed using MetaMorph acquisition/analysis software (Universal Imaging Corp., Downingtown, PA, USA).

The amount of mitochondrial fragmentation was evaluated using the Shape Factor function of the MetaMorph software. Shape Factor evaluates the circular shape of an object and attributes a score near 0 in the case of a flattened object and a score near 1 for a perfect circle. We defined three shape factor groups to classify the different morphologies of the mitochondrial networks: Group I: shape factor 0–0.3 consisted of cells with a filamentous mitochondrial network; Group II: shape factor 0.3–0.6 consisted of cells with filamentous network but with the presence of round-shaped mitochondria; and Group III: shape factor 0.6–1 consisted of cells with high fragmentation with only round-shaped mitochondria. For each cell type, a total of 100 images, collected in four different experiments, were used to calculate the percentage of the different shape factors.

### ATP evaluation

Fibroblasts were plated at 8000 cells/well in 96-well plates. The next day, total cellular ATP was measured using the ATPlite kit (PerkinElmer Life Sciences) according to the manufacturer's procedure. This method is based on the mono-oxygenation of luciferin, which is catalysed by luciferase in the presence of Mg^2 +^, ATP, and oxygen, resulting in a luminescent signal that is proportional to the ATP concentration. The plate was read on a Victor plate reader (Perkin Elmer) in luminescence mode. The cell numbers were evaluated with the CyQUANT kit (Invitrogen). Total ATP was then normalised for cell number.

### Statistical analyses

The data, except where otherwise indicated, are reported as the mean +/− SD values or as representative of at least three independent experiments with similar results. Statistically significant differences between controls and patients were determined in all the experiments by one-way ANOVA analyses with Bonferroni's post-test and by Student's *t*-test; *, ** and *** indicated p < 0.05, p < 0.01 and p < 0.001, respectively. A p value < 0.05 was considered statistically significant. Both analyses gave similar significance except where specified.

## Results

### PKAN fibroblasts show altered iron dependent oxidative status

**Fig. 1:**
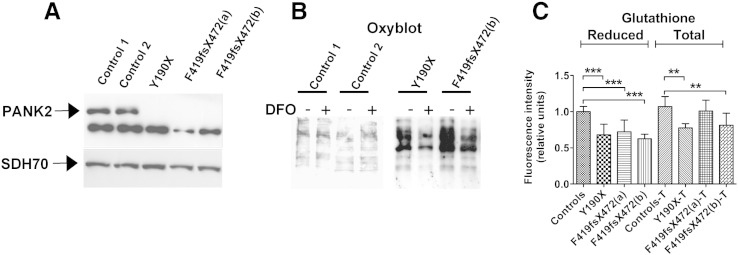
PKAN fibroblasts show altered oxidative status. (A) The determination of the PANK2 protein content in fibroblast soluble extracts. The arrows point to PANK2 mature peptide (47 kDa) and to SDH70 used as loading control. The other lower band, present in patients and controls, is a non-specific reaction of the antibody. (B) The fibroblasts were treated or not with 100 μM deferoxamine (DFO) for 18 h, and the levels of carbonylated proteins in soluble cell extracts were then analysed by Oxyblot. One representative of three independent experiments is shown. (C) Measurements of total and reduced glutathione content in fibroblasts evaluated using the specific fluorescent probe ThiolTracker Violet. The images were acquired by fluorescence microscopy, and the quantified signal, relative to controls, was plotted as the mean and standard deviation of three independent experiments in triplicate. **p < 0.01, ***p < 0.001.

We analysed fibroblasts from three PKAN patients and healthy subjects. One PKAN patient carried a homozygous c.569insA mutation that resulted in a premature stop codon at amino acid position 190 (p. Y190X) (Hartig et al., 2006); the other two patients are brothers and carried a homozygous c.1259delG mutation, producing a frame shift mutation resulting in the substitution of 53 amino acids and the creation of a stop codon (F419fsX472) (Campanella et al., 2012, Zorzi et al., 2011). As shown by western-blot analysis in Fig. 1A, PANK2 is virtually absent in all three patients, whilst a normal level of the protein is present in the controls. We then determined cellular oxidative status and its relationship with iron content in these cells by treating control and PKAN fibroblasts with the iron-chelator deferoxamine (DFO) for 18 h. The content of carbonylated proteins in the DFO-treated and untreated cellular extracts was then evaluated by Oxyblot (Fig. 1B). The results confirmed that untreated PKAN fibroblasts showed increased carbonylated protein levels compared to control cells (Fig. 1B, DFO −). Interestingly, whilst DFO treatment ameliorated the level of carbonylated proteins in PKAN fibroblasts, it was completely ineffective in control fibroblasts (Fig. 1B, DFO +). Moreover, by monitoring ThiolTracker Violet intensity, we observed a significant reduction in levels of both total and reduced glutathione in PKAN fibroblasts compared to controls (Fig. 1C).

### PKAN fibroblasts show mitochondrial iron homeostasis dysfunction

**Fig. 2:**
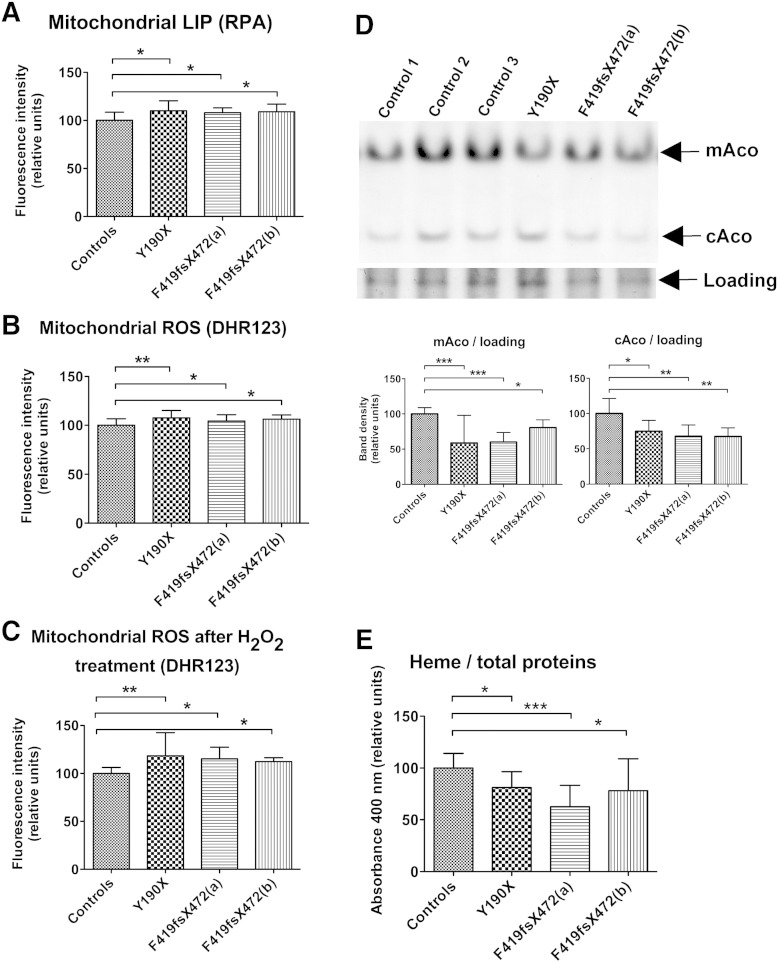
PKAN fibroblasts show altered mitochondrial iron handling. (A) The evaluation of the mitochondrial LIP in untreated fibroblasts by the specific probe RPA. The mean and SD of three independent experiments made in quadruplicate, and the bars indicate statistically significant differences, * p < 0.05. (B and C) The cells were treated or not with 0.3 mM H_2_O_2_ for 30 min, and the mitochondrial ROS generation was evaluated on untreated B or treated C fibroblasts using the fluorescent probe DHR-123. The mean and SD of three independent experiments made in quadruplicate, *p < 0.05, **p < 0.01. (D) The enzymatic activity of aconitase was evaluated on soluble cell homogenates that were separated on non-denaturing PAGE gels. The arrows indicate the position of mitochondrial aconitase (mAco), of cytosolic aconitase (cAco) and of the protein band stained with Coomassie blue used for loading control. The histograms show the densitometry of aconitase bands relative to controls, with the mean and SD of three independent experiments, *p < 0.05, **p < 0.01, ***p < 0.001. (E) The evaluation of heme content in untreated fibroblasts by absorbance at 400 nm of the soluble cell lysates. The mean and SD of six independent experiments, *p < 0.05, ***p < 0.001.

To verify whether the defect in PANK2 activity affected mitochondrial iron homeostasis, we tested for the presence of potentially toxic free iron (LIP) in the mitochondrial compartment using the iron sensing mitochondrial target-specific fluorescent probe (rhodamine B-[(1,10-phenanthrolin-5-yl)-aminocarbonyl]benzyl ester, RPA). This probe has been largely used with success in previous works to specifically measure the mitochondrial LIP (Cabantchik, 2014, Ma et al., 2015, Rauen et al., 2007). Rhodamine B-[(phenanthren-9-yl)-aminocarbonyl]-benzylester (RPAC), an analog of RPA but without iron binding capacity and subsequent quenching, was used to monitor the incorporation of the probe driven by mitochondrial-membrane-potential in the control and patients' cells. The cells were incubated with or without 100 mM ferric ammonium citrate (FAC) for 18 h. The RPAC fluorescence was measured and the results indicated that the amount of incorporated probe was similar in control and patients' cells in all the conditions (not shown). Then, RPA fluorescence was measured before and after the addition of the iron chelator PIH (pyridoxal isonicotinoyl hydrazone) (Rauen et al., 2007). The result indicated that the size of the mitochondrial LIP was statistically higher in PKAN fibroblasts than in control cells in both basal growth conditions (Fig. 2A) and with iron supplementation (not shown). As labile iron can promote ROS formation, we measured their generation using the ROS sensitive mitochondrial probe dihydrorhodamine 123 (DHR-123) in basal growth condition and after iron supplementation (Campanella et al., 2009). The fluorescence intensity was significantly higher in PKAN fibroblasts than in controls under basal growth conditions (Fig. 2B) and after iron addition (not shown). In PKAN cells only, this difference further increased after incubation with 0.3 mM H_2_O_2_ both in basal growth condition (Fig. 2C) and after iron supplementation (not shown). To test the efficiency of ISC biosynthesis, we evaluated the in-gel enzymatic activity of cAco and mAco, two ISC-containing proteins, after separation on non-denaturing PAGE (Fig. 2D, upper panel), a higher sensible method with respect to the one previously used (Campanella et al., 2012). Densitometric analysis of the bands showed a reduced enzymatic activity of both aconitase forms (Fig. 2D, lower panels). However, the amount of aconitase proteins as evaluated by western blotting with specific antibodies was similar between PKAN and control fibroblasts (not shown). Furthermore, we evaluated heme biosynthesis that, other than depending on the ISC-containing ferrochelatase, represents the other major iron-containing product in mitochondria. We measured heme content in fibroblasts by monitoring the absorbance of soluble extracts from cells lysed in formic acid at 400 nm. We observed a significant difference in heme content, corresponding to a 25% reduction in PKAN compared to the control fibroblasts (Fig. 2E).

### PKAN fibroblasts show energetic mitochondrial dysfunction

**Fig. 3:**
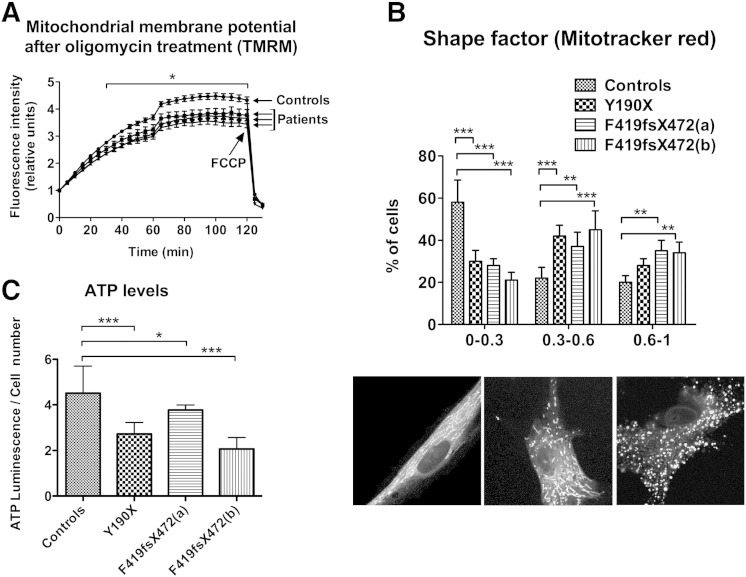
PKAN fibroblasts show altered mitochondrial functionality. (A) The fibroblasts were incubated with 4 μg/ml of oligomycin for 1 h, and the mitochondrial-specific fluorescent probe TMRM was then added. Its incorporation was followed using the IN-Cell Analyzer 1000 system for 2 h. Plots, normalised on starting points, show the kinetics of TMRM incorporation, with the mean and SEM of three independent experiments, * p < 0.05 from 30 min with the exception of patient Y190X that resulted significant only from 60 min by one-way ANOVA analyses with Bonferroni's post-test. The arrow indicates the time of FCCP addition. (B) Fibroblasts were incubated with MitoTracker Red fluorescent probe. The evaluation of the mitochondrial shape factor was performed on images obtained by fluorescence microscopy. The histogram shows the percentage of cells with the indicated shape factor, with the mean and SD of four independent experiments, **p < 0.01, ***p < 0.001. (C) Evaluation of ATP content in fibroblasts. Total ATP levels were evaluated by the ATPlite kit. The data are presented as the mean of two controls (reported as a single bar) and three patients, reported separately, with the mean and SD of three independent experiments. *p < 0.05, ***p < 0.001.

We further investigated the ability of mitochondria to maintain the cellular energy requirement. The mitochondrial membrane potential was evaluated with the mitochondria-specific fluorescent probe tetramethylrhodamine methyl ester (TMRM) on fibroblasts in the presence of the ATPase inhibitor oligomycin. The kinetics of TMRM incorporation was followed for 2 h by monitoring the fluorescence of the probe using the IN-Cell Analyzer 1000 system. Starting from 30 min, the mean kinetics of the controls was statistically higher than for PKAN patient fibroblasts (Fig. 3A, *p < 0.05). These data indicated that the PKAN fibroblasts incorporated less probe compared to control cells, suggesting lower membrane hyperpolarisation. We investigated mitochondrial morphology after loading cells with Mitotracker Red and examination by fluorescence microscopy. The fibroblasts were scored into different categories on the basis of mitochondrial morphology. Representative images of these classes of mitochondrial morphology are shown in Fig. 3B. A total of 100 different images were obtained and quantified for each sample. The amount of mitochondrial fragmentation was evaluated using the Shape Factor function of MetaMorph software. This function evaluates the circular shape of an object and attributes a score near 0 in the case of a flattened object and a score near 1 for a perfect circle. The Shape Factor average of PKAN patients' fibroblasts was closer to 1 than in the control subjects. The mitochondrial network of PKAN patients with loss-of-function mutations was more fragmented than controls, indicating an impaired bioenergetic profile. The difference between these two samples was statistically significant (Fig. 3B upper panel; *p < 0.05; **p < 0.01). In line with this observation, we observed a reduction in ATP levels in PKAN fibroblasts compared to control cells (Fig. 3C; *p < 0.05; **p < 0.01).

### Generation and characterisation of induced neurons from PKAN fibroblasts

**Fig. 4:**
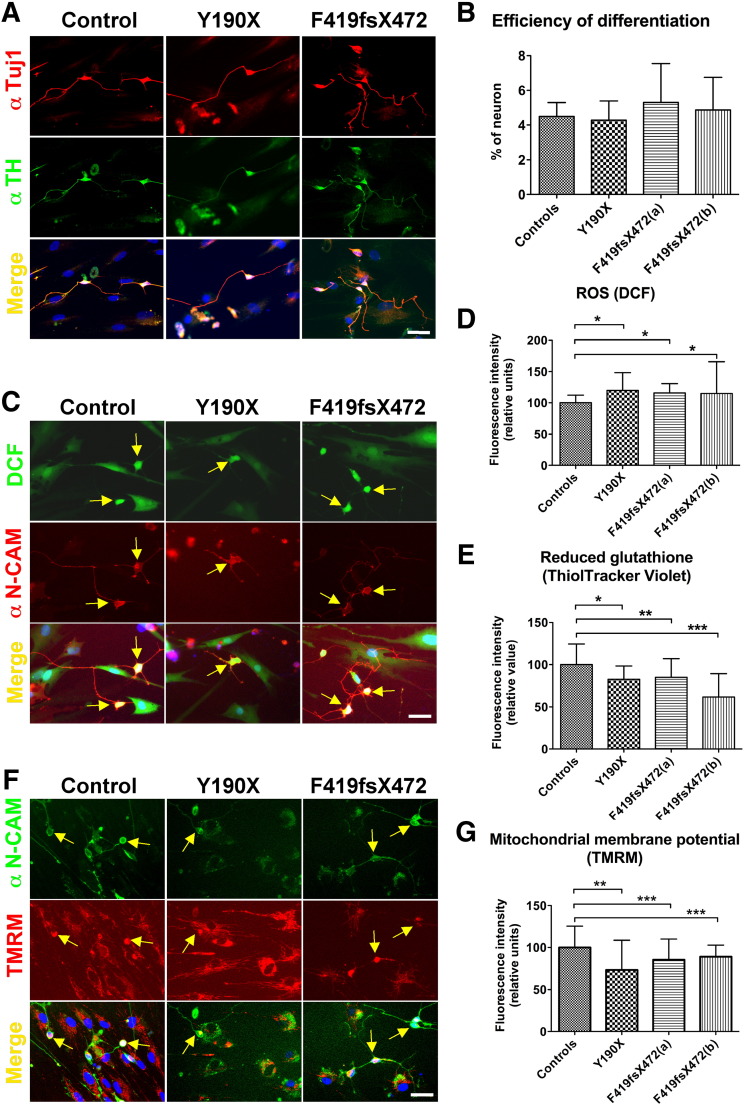
Characterisation of induced neurons obtained from PKAN fibroblasts by direct reprogramming. (A) The induced neurons were stained for neuronal class III beta tubulin (α-Tuj1, top panels in red) and tyrosine hydroxylase (α-TH, middle panels in green). Hoechst was used to stain cell nuclei (in blue), and the lower panels show the merged images, scale bar = 100 μm. Tuj1, a neuronal marker, was used to calculate the reprogramming efficiency, and the results are plotted in panel B. (C) Live cells stained with the ROS-sensing fluorescent probe DCF (top panels in green) and with the neuron-specific anti-N-CAM antibody (middle panels in red); merged images are shown in the lower panels, with the nuclei stained with Hoechst. The fluorescence was analysed using the IN Cell Analyser 1000 System, scale bar = 100 μm. (D) The DCF fluorescence signal from N-CAM-positive cells (from panel C) were quantified, and the data are shown in the plots as the mean and SD of three independent experiments (at least 50 neurons per type), *p < 0.05. (E) The ThiolTracker Violet fluorescence signal from N-CAM-positive cells was quantified (experiments were performed as in panel C, with ThiolTracker in place of DCF), and the data are shown in the plots as the mean and SD of three independent experiments (at least 50 neurons per type), *p < 0.05, **p < 0.01. (F) Live cells were stained with the mitochondrial membrane potential sensible fluorescent probe TMRM (middle panels in red) and with the neuron-specific α-N-CAM antibody (top panels in green). The merged images are shown in the lower panels, with nuclei stained with Hoechst. The fluorescence was analysed using an IN-cell analyser, scale bar = 100 μm. (G) The TMRM fluorescence signal from N-CAM-positive cells (from panel F) was quantified, and the data are shown in the plots as the mean and SD of three independent experiments (at least 50 neurons per type), **p < 0.01, ***p < 0.001.

Human neurons were generated from patient and control fibroblasts by direct neuronal reprogramming (Caiazzo et al., 2011, Cozzi et al., 2013). Lentiviruses expressing the transcription factor combination Ascl1, Nurr1 and Lmx1a were used to transduce fibroblasts to generate iNs, which were then maintained in a neurobasal medium supplemented with a cocktail of growth factors and inducing molecules (see the Material and methods section). iNs were identified in the reprogrammed cell culture by immunostaining with the specific neuronal marker class III β-tubulin (TuJ1), N-CAM and MAP2, as well as the dopaminergic-specific enzyme tyrosine hydroxylase (TH) (Fig. 4A and data not shown). At least 50% of the TuJ1-positive cells were also positive for TH. We used TuJ1-positive cells to evaluate neuronal reprogramming efficiency, which was approximately 5% and comparable between PKAN and control cells (Fig. 4B). We then used iNs to evaluate several parameters, including the oxidative status and mitochondrial functionality. Due to the low efficiency of direct cellular reprogramming, we developed a method for in vivo analysis at single cell level by selecting the live neurons using the specific neuronal membrane marker N-CAM.

### PKAN induced neurons show altered oxidative status

First, we analysed the amount of ROS specifically present in iNs grown in basal conditions. The cells were incubated with the fluorescent ROS-sensitive dichlorofluorescein (DCF), and the images were acquired by the IN-Cell Analyzer 1000 system. The identity of iNs was verified by the expression of the neuronal specific marker N-CAM, and only the N-CAM-positive cells were utilised to calculate the fluorescence of the DCF (Fig. 4C). The quantification of fluorescent intensity specific to iNs indicated that PKAN iNs contained higher ROS levels than control iNs (Fig. 4D). Moreover, an analysis of ThiolTracker Violet treated cells revealed that PKAN iNs contained significant lower reduced glutathione levels compared to control iNs (Fig. 4E).

### PKAN induced neurons show alterations in mitochondrial membrane potential

A similar approach was used to evaluate mitochondrial functionality in neuronal cells. We measured mitochondrial membrane potential by quantifying the TMRM signal 15 min after treatment with the probe; the images were collected as above. As previously described, only N-CAM-positive cells were utilised to calculate the TMRM fluorescence (Fig. 4F). The quantification of TMRM intensity showed that PKAN iNs had significantly lower membrane potentials compared to control iNs (Fig. 4G).

## Discussion

PKAN is a severe and disabling neurodegenerative disorder, the pathogenic mechanisms of which are largely unknown (Zhou et al., 2001). The low amount of CoA due to the PANK2 enzymatic defect has a devastating impact on many biological processes. CoA is utilised as a cofactor in approximately 4% of all known enzymes; thus, its imbalance inhibits a wide range of enzymatic pathways (Leonardi et al., 2005). This effect was highlighted by the studies of PKAN disease models in flies and mice. These analyses revealed different types of CoA-deficiency related phenotype, such as the following: sterility (Afshar et al., 2001, Kuo et al., 2005); abnormal locomotor function, neurodegeneration, and reduced life span (Bosveld et al., 2008); mitochondrial impairment (Brunetti et al., 2012, Rana et al., 2010); altered cytoskeleton function (Siudeja et al., 2012); protein acetylation (Siudeja et al., 2011); disrupted circadian locomotor patterns and a unique transcriptional signature (Pandey et al., 2013). However, all of these investigations have not clarified the mechanism of one of the major features of the human disease, brain iron accumulation, which has not been reported neither in flies nor in mice (Brunetti et al., 2012, Rana et al., 2010).

To approach this question, we previously examined iron metabolism in PKAN fibroblasts, demonstrating an abnormal behaviour of *PANK2* deficient cells in response to treatment with iron and the consequent alteration of oxidative status (Campanella et al., 2012). In the previous study, we concentrated our attention on cytosolic iron handling. As mitochondrial iron homeostasis and cytosolic iron homeostasis are strictly interrelated, we hypothesised that iron mishandling could also occur in the mitochondria, thereby affecting organelle functionality. For this reason, we extended our analysis to mitochondria to determine whether and how mitochondrial iron alteration could impact on the cell's bioenergetic profile. Interestingly, we detected a larger mitochondrial LIP also under basal conditions, whereas the increase in the size of the cytosolic LIP was appreciable only after chronic iron supplementation (Campanella et al., 2012). Nevertheless, the availability of high levels of mitochondrial iron did not appear to be properly utilised in ISC and heme biosynthesis, thus they resulted defective in PKAN fibroblasts. The reasons why this occurs and how the PANK2 deficiency is involved are still obscure. Very recent data, obtained on isolated mammalian mitochondria, demonstrated that ISC biosynthesis requires, other than iron and sulphur, also GTP, NADH and ATP (Pandey et al., 2015). A very reliable hypothesis could be that the impairment of the Krebs cycle due to CoA deficiency might result in lower production of GTP and NADH, with the consequent partial iron utilisation in ISC biosynthesis.

Moreover, LIP and ROS are tightly controlled in cell and large variations are not expected. In fact, the detected increased size of the LIP resulted in a slight increase in mitochondrial ROS production, which plays a central role in the regulation of ISC enzyme activity and, in particular, in Acos enzyme functions. Both the cAco and mAco contain [4Fe–4S] clusters that are responsible for their catalytic activity, which is regulated by reversible oxidation (Bulteau et al., 2003). The main role of mAco is to control cellular ATP production via the regulation of intermediate flux in the Krebs cycle. cAco has two different functions: in the ISC-containing form, it operates as an enzyme, whereas in the oxidised form, it is involved in the control of iron–protein translation as IRP1 (Lushchak et al., 2014). Thus, the reduction of their activities, caused by their higher oxidation produced by ROS or by diminished production of ISC, has an effect both on ATP production and iron homeostasis, which we demonstrated to be altered in PKAN fibroblasts.

In addition, other mitochondrial features appeared defective in patients' cells, such as the mitochondrial membrane potential and mitochondrial morphology, suggesting a global organelle impairment, which can lead to energetic failure. Interestingly, a mitochondrial morphology alteration was also recently reported in the muscles of a PKAN patient with a defined genetic mutation (Brunetti et al., 2014), indicating that variation of mitochondrial morphology can also be detected in vivo in different patient cell types. An altered mitochondrial membrane potential was also detected in neurons derived from a *Pank2* KO mouse model (Brunetti et al., 2012).

Obviously, we cannot exclude the possibility that the energetic impairment detected in PKAN fibroblasts could also derive from membrane alterations due to CoA deficiency-induced defects in phospholipid synthesis; more detailed studies are needed to clarify this point. In fact, the partial mitochondrial swelling and depolarisation detected in PKAN fibroblasts suggest mitochondrial membrane damage. However, as reported by previous studies on isolated rat liver mitochondria, iron may catalyse the peroxidation of lipids in the mitochondrial inner membrane, which can result in a limited depolarisation (Gall et al., 2012). Mitochondrial membrane depolarisation is a strong stimulus for opening the mitochondrial permeability transition pore (Bernardi et al., 1992, Gogvadze et al., 2003), which is considered one of the causative events of neuronal injury (Crompton, 2004). The opening of the pore may facilitate mitochondrial iron import from the cytosolic compartment, further increasing the size of the mtLIP, not utilised by the intra-mitochondrial biosynthetic pathways, thus exacerbating the injury.

Extensive studies on *Saccharomyces cerevisiae* demonstrated that the cells respond to reduced ISC synthesis with increased cellular iron uptake and intracellular iron re-distribution, resulting in mitochondrial iron accumulation until the metal precipitates as an amorphous mineral (Lill and Muhlenhoff, 2008). These pathologic events have also been described to occur in human disorders, such as Friedreich's ataxia (Koeppen et al., 2007, Vaubel and Isaya, 2013) and Parkinson disease (Horowitz and Greenamyre, 2010a, Mastroberardino et al., 2009) as well as during the physiological ageing process (Isaya, 2014). In fact, in recent times, there has been growing evidence of the role of mitochondrial iron dysfunction in the pathogenesis of common neurodegenerative diseases (Horowitz and Greenamyre, 2010b). Overall, the data indicate that defects in mitochondrial ISC biogenesis seem to represent a common primary mechanism, which triggers abnormal intracellular iron distribution. This effect results in cytosolic iron starvation and mitochondrial iron accumulation, oxidative phosphorylation deficits and oxidative stress (Isaya, 2014, Nunez et al., 2012).

To clarify whether this abnormal iron distribution has a primary or a secondary etiopathological role or is simply an epiphenomenon, more work on different disease models is necessary. In any case, even if iron dysregulation in PKAN may play a secondary role, its accumulation is harmful and can aggravate the damage observed in the basal ganglia, thus possibly justifying the iron chelation treatment proposed in patients (Zorzi et al., 2011). Certain brain regions, such as the globus pallidus and the substantia nigra, have high iron contents, which are necessary for the synthesis of neurotransmitters. These regions are particularly prone to the process of abnormal intracellular iron distribution, which may also contribute to the age-dependent iron accumulation (Isaya, 2014, Urrutia et al., 2013). We approached this question employing direct neuronal cell reprogramming technology (Amamoto and Arlotta, 2014, Caiazzo et al., 2011), which allowed us to obtain human neurons starting from fibroblasts. Although this technique has an overall low efficiency in neuronal generation, we succeeded in obtaining enough neurons from PKAN and healthy subject fibroblasts for an in depth analysis. Our data indicate that it is feasible to generate human neuronal cells, even in cases of genetic alterations that lead to mitochondrial impairment, as in the case of PKAN. In addition, as previously determined in fibroblasts, our results confirm, for the first time in PKAN induced neurons, the presence of an altered oxidative status in basal conditions and the impairment of mitochondrial functions. iN technology represents a very powerful system to investigate disease-relevant pathogenetic mechanisms. However, this technology does not currently allow the generation of striatal neurons, which represent the most affected neuronal population in PKAN. Considering how fast direct neuronal reprogramming technologies have been developing, we predict that an even more appropriate human neuronal model will be soon available to further investigate PKAN pathogenetic mechanisms. Nevertheless, our approach has enabled the identification of altered biological processes that are relevant in diseased neurons; therefore, the present results are more relevant than the analyses of other cell types. Our results indicate that PANK-iNs might also represent a suitable system for establishing medium-scale drug screenings to identify new candidate therapeutic compounds for this disease.

## Conflict of interested statement

None declared.
